# Biclustering for the comprehensive search of correlated gene expression patterns using clustered seed expansion

**DOI:** 10.1186/1471-2164-14-144

**Published:** 2013-03-05

**Authors:** Taegyun Yun, Gwan-Su Yi

**Affiliations:** 1 Department of Information and Communications Engineering, KAIST, 291 Daehak-ro, Yuseong-gu, Daejeon, 305-701, Republic of Korea; 2 Department of Bio and Brain Engineering, KAIST, 291 Daehak-ro, Yuseong-gu, Daejeon, 305-701, Republic of Korea

## Abstract

**Background:**

In a functional analysis of gene expression data, biclustering method can give crucial information by showing correlated gene expression patterns under a subset of conditions. However, conventional biclustering algorithms still have some limitations to show comprehensive and stable outputs.

**Results:**

We propose a novel biclustering approach called “BIclustering by Correlated and Large number of Individual Clustered seeds (BICLIC)” to find comprehensive sets of correlated expression patterns in biclusters using clustered seeds and their expansion with correlation of gene expression. BICLIC outperformed competing biclustering algorithms by completely recovering implanted biclusters in simulated datasets with various types of correlated patterns: shifting, scaling, and shifting-scaling. Furthermore, in a real yeast microarray dataset and a lung cancer microarray dataset, BICLIC found more comprehensive sets of biclusters that are significantly enriched to more diverse sets of biological terms than those of other competing biclustering algorithms.

**Conclusions:**

BICLIC provides significant benefits in finding comprehensive sets of correlated patterns and their functional implications from a gene expression dataset.

## Background

Genes in common regulatory mechanisms under specific conditions are likely to show similar expression patterns. Identifying those patterns and the corresponding genes is one of the most important steps of microarray analysis to reveal the novel functions of genes, transcription factor-target relationships, and concerted gene functions in pathogenesis [1-3]. Clustering analysis is commonly performed to identify groups of genes expressed in similar patterns. However, an accurate gene expression analysis can be hindered owing to limitations in clustering analysis. Most clustering algorithms try to find non-overlapping groups of genes that show similar expression patterns under all experimental conditions. In a common situation, genes tend to be co-regulated, and thus, they could be co-expressed under a subset of experimental conditions, but not under all conditions. Parts of genes in one expression pattern may exhibit a different expression pattern under other conditions because genes can participate in more than one function differently depending on the specific conditions [4]. To resolve this issue, a biclustering method can suitably substitute general clustering methods by providing correlated gene clusters under a subset of conditions in an unsupervised gene expression analysis.

A bicluster can be defined as a sub-matrix in a whole gene expression data matrix representing groups of genes that show coherent expression patterns under a subset of conditions [5]. It is required to search exhaustive sets of biclusters for functional analysis of gene expression dataset. However, extracting complete sets of biclusters from a whole microarray data matrix is an NP-hard problem that requires massive computation [6]. To avoid computational issues in biclustering, most existing biclustering algorithms use a greedy iterative heuristic approach that locally improves an appropriate scoring function starting from initial seed biclusters. To search more comprehensive sets of meaningful biclusters with a greedy iterative heuristic biclustering approach, it is important to determine initial seed biclusters and score functions properly.

The output from conventional biclustering methods shows lack of stability. Since common biclustering methods depend on random starting seeds, the numbers and the contents of resulting biclusters are changing every time even though the biclustering algorithm is applied to the same microarray datasets. Moreover, random starting seeds cannot guarantee diverse searching of biclustering and coherent biclustering results. However, in conventional biclustering methods, the use of random starting seeds was inevitable choice to compromise the computation complexity and there have been a few studies to overcome this limitation. Erten and Sözdinler [7] proposed a localization method that reorders rows and columns in an initial data matrix to exhibit similar patterns in nearby locations. Although this method could alleviate a part of the random seed issue by raising a chance to extract biclusters with similar patterns in a localized matrix, it could not solve comprehensiveness issues of random seeds.

The way to set the scoring function of bicluster is also important to improve the performance of biclustering. Mean squared residue, which measures variability of biclusters based on the arithmetic mean of gene expression, was the first scoring function used to find biclusters [8] and it was used in several other biclustering methods subsequently [9-11]. Mean square residue is a fundamental measure to find similar expression values, however, this measure is not adequate for finding the scaling patterns of biclusters as proved by Aguilar-Ruiz [12]. Inability to find a scaling pattern can be a major drawback in biclustering analysis because groups of genes showing similar expression patterns with different scales are also meaningful correlated gene clusters that we aim to find.

In this point of view, the correlation coefficient can be an alternative scoring function to the mean squared residue. With this measure, correlated expression patterns, including both shifting and scaling patterns, can be detected and this is more relevant to the purpose of biclustering to find the co-expressed gene clusters under the same biological regulation. Allocco *et al.*[13] showed that if the correlation coefficient of two genes is greater than 0.84, there is more than 50% probability that such genes are regulated by a common transcription factor. Bhattacharya and De [14] proved that the correlation coefficient-based biclustering method, Bi-Correlation Clustering Algorithm (BCCA), can find a greater number of common transcription factors and a significantly enriched biological function term than other non-correlation-based biclustering methods.

Several correlation-based biclustering approaches have recently been proposed [15-19]. BCCA is a Pearson correlation coefficient-based biclustering method that finds groups of genes showing a correlated expression pattern across a subset of microarray conditions. The process of BCCA begins with pairs of genes. It backwardly eliminates uncorrelated conditions for each selected pair of genes to find correlated sets of biclusters. Theoretically, a large number of biclusters can be found with BCCA since BCCA searches biclusters from all pairs of correlated genes. However, BCCA is unable to extract comprehensive sets of biclusters in real situations since a backward elimination approach limits search spaces. Bozda˘ g *et al.*[15] proposed the Correlated Pattern Biclusters (CPB) algorithm, which discovers biclusters by setting reference genes with randomly selected columns, and then adding rows with high correlation and determining columns that have a smaller Root Mean Squared Error. In this case, the search space can be restricted again by the randomly selected seeds of columns. Ayadi *et al.*[19] proposed the Pattern-Driven Neighborhood Search (PDNS) algorithm for finding correlated expression patterns of biclusters based on Spearman’s rank correlation. It converts an original numerical matrix to a discretized matrix with −1, 0, or 1 for having trajectory patterns of genes. By using an initial solution of biclusters with a discretized matrix, this algorithm locally improves a solution by using descent search and perturbation. Because the PDNS algorithm requires initial solutions of biclusters from random selection or other fast greedy algorithms, such as Cheng and Church algorithm, biclustering results can be varied by selection of initial biclusters. The Qualitative Biclustering algorithm (QUBIC) is a recently proposed gene-wise discretization-based biclustering algorithm to solve the general form of the biclustering problem efficiently, including constant, shifting, and scaling patterns [20]. QUBIC converts a microarray data matrix into a simplified integer matrix called a representing matrix, from which it finds biclusters. Therefore, QUBIC may not identify subtle changes of expression patterns. In addition, the search space in QUBIC is limited by the discretization process.

In this paper, we propose a novel biclustering algorithm called BIclustering by Correlated and Large number of Individual Clustered seeds (BICLIC) aiming to search comprehensive sets of biclusters with correlated gene expression patterns. The primary process of BICLIC is not conducted with random seed biclusters, but with the full search of correlated seed bi-clusters that are determined by individual dimension-based clustering. Then comprehensive sets of correlated seed biclusters are expanded to larger biclusters using a greedy iterative heuristic approach with the Pearson correlation coefficient as the scoring function. As a result, BICLIC can find comprehensive biclusters accurately and also provides stable output in multiple runs.

We demonstrate that our proposed BICLIC method outperforms other conventional biclustering methods in finding correlated gene expression patterns both in simulated data sets and in real microarray datasets.

## Results and discussion

The proposed BICLIC algorithm is implemented in the R language. R-code of the BICLIC algorithm is freely available from http://bisyn.kaist.ac.kr/software/biclic.htm.

In this section, the performance of our biclustering algorithm will be compared with those of three well-known existing bicluster algorithms: BCCA, CPB, and QUBIC. The BCCA, CPB, and QUBIC programs are from each paper’s cited sources. The performance comparison can be divided into two parts. In the first part, simulated datasets are used to test the accuracy and the coverage of the biclustering algorithm to identify implanted biclusters that have various correlated patterns. In the second part, a real microarray dataset is used to show that BICLIC can extract more diverse sets of correlation-based biclusters than those extracted by compared methods, BCCA and QUBIC, and the extracted biclusters from BICLIC are significantly enriched in biological terms, such as the gene ontology (GO) functional category [21] and the KEGG pathway [22].

### Simulated datasets

The purpose of this test is to verify the ability of BICLIC to search comprehensive correlated patterns as well as to compare the performance of BICLIC with that of the BCCA and QUBIC algorithms. BCCA is a correlation-based biclustering algorithm, whereas QUBIC is known for its ability to detect various patterns of biclusters, including correlation patterns. BICLIC can find diverse sets of correlated patterns, such as shifting, scaling, and shifting-scaling patterns. Shifting and scaling patterns are defined in [12]. In a shifting pattern, each column is shifted by an additive factor. A shifting pattern follows equation 4.

(4) $$e_{\mathrm{ij}}=\pi_{i}+\beta_{j}$$

The expression of the *i*th gene in the *j*th condition, *e*_*ij*_, is a shifted expression of a base expression *π* in the *i*th row shifted by a shifting factor *β* in the *j*th column. In a scaling pattern, each column is scaled by multiplicative factors. A scaling pattern follows equation 5.

(5) $$e_{\mathrm{ij}}=\pi_{i}\times \alpha_{j}$$

The expression in the *i*th gene in the *j*th condition, *e*_*ij*_, is a scaled expression of a base expression *π* in the *i*th row by scaled by a scaling factor *α* in the *j*th column. The shifting-scaling pattern is a combination of a shifting pattern and a scaling pattern. Each expression is shifted by a shifting factor and scaled by a scaling factor. The shifting-scaling pattern follows equation 6.

(6) $$e_{\mathrm{ij}}=\pi_{i}\times \alpha_{j}+\beta_{j}$$

Bozda˘ g proved that the value of the Pearson correlation coefficient is 1 for a perfect shifting, scaling, and shifting-scaling pattern [23]. Therefore, any correlated patterns of shifting, scaling, and shifting-scaling patterns can be extracted by the BICLIC biclustering method, which has the Pearson correlation coefficient as its scoring function. BICLIC considers positively correlated patterns when it generates biclusters because it collect genes with positively correlated with seed bicluster. However, negatively correlated patterns also can be discovered when positively correlated biclusters are compared each other and negatively correlated biclusters exists.

To simulate each correlated pattern, a 1000 X 100 data matrix is generated with random values in a normal distribution whose mean is 0 and standard deviation is 1. For each type of correlated pattern, 10 data matrices are generated, resulting in a total of 30 data matrices. For each data matrix, 10 non-overlapping biclusters of size 100 X 10 are implanted in the matrix. Shifting, scaling, and shifting-scaling patterns of biclusters are generated from equations 4, 5, and 6, respectively. Shifting and scaling factors are randomly generated from a normal distribution whose mean is 0 and standard deviation is 1. To generate positively correlated patterns, randomly generated scaling factors are changed to absolute values of the original random values.

In addition, simulated datasets that have implanted biclusters with different-sized columns are generated to study the effect of column size on the performance of the biclustering algorithms. The size of the whole data matrix is 1000 X 100, the same as that of the previous simulated dataset. The number of rows of a bicluster is fixed as 100, but the number of columns varies from 20 to 100. Five different sized biclusters are implanted in each 1000 X 100 data matrix. These simulated datasets are also generated for three kinds of correlated patterns: shifting, scaling, and shifting-scaling.

To compare the accuracy of different biclustering algorithms on simulated datasets, the average match score proposed by Prelic *et al.*[24] is used. The average match score is defined in equation 7.

(7) $$S_{G}\left(M_{1},M_{2}\right)=\frac{1}{\left|M_{1}\right|}\sum_{\left(G_{1},C_{2}\right)\in M_{1}}\max_{\left(G_{2},C_{2}\right)\in M_{2}}\frac{\left|G_{1}\cap G_{2}\right|}{\left|G_{1}\cup G_{2}\right|}$$

**Figure 1:**
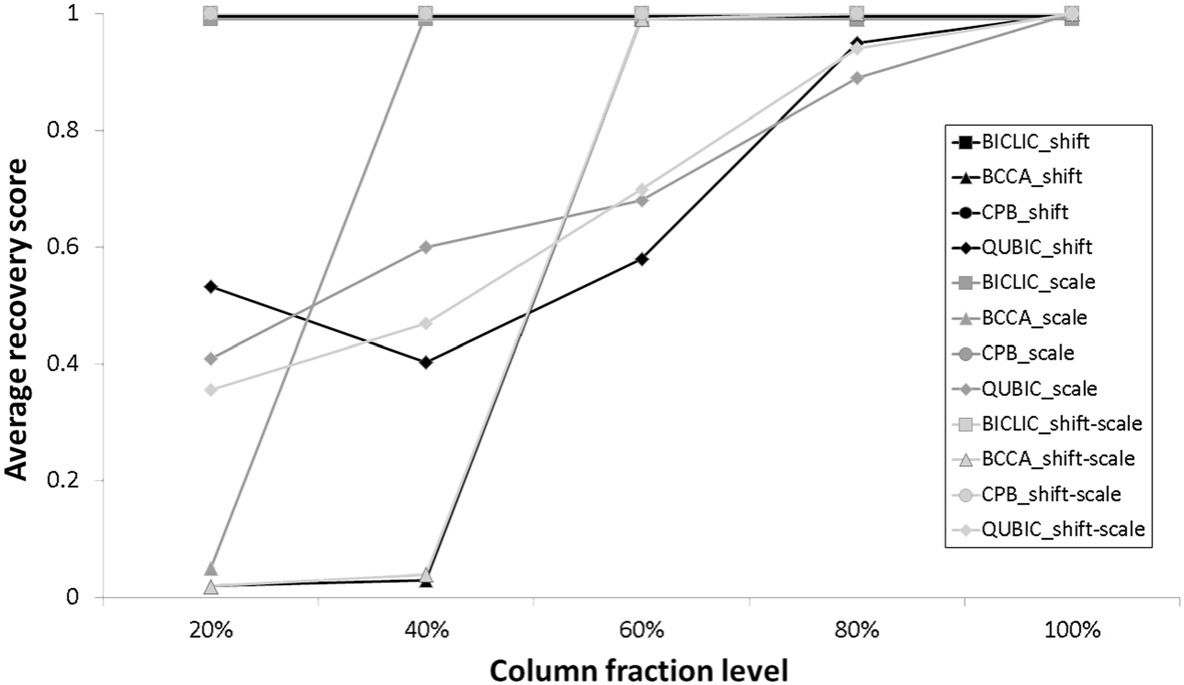
**Effect of column fraction level on average recovery score in shifting, scaling, and shifting-scaling pattern.** Each average recovery score is the mean value of average recovery scores from 10 independent datasets.

*G*_*1*_ and *G*_*2*_ are gene sets in a bicluster set *M*_*1*_ and *M*_*2*,_ respectively. |*G*_*1*_∩*G*_*2*_| is the number of data elements in the intersection of *G*_*1*_ and *G*_*2*_ and |*G*_*1*_∪ *G*_*2*_| is the number of data elements in the union of *G*_*1*_ and *G*_*2*_. *S*_*G*_(*M*_*1*_, *M*_*2*_) represents the average of the maximum match score for all biclusters in *M*_*1*_ when compared to biclusters in *M*_*2*_. If *M*_*1*_ is the set of implanted true biclusters and *M*_*2*_ is a set of generated biclusters, *S*_*G*_(*M*_*1*_,*M*_*2*_) represents the average recovery score. The average recovery score measures how well the biclustering algorithm recovers implanted true biclusters. Conversely, if *M*_*1*_ is the set of generated biclusters and *M*_*2*_ is the set of implanted true biclusters, the average match score, *S*_*G*_(*M*_*2*_, *M*_*1*_), represents the average relevance score. The average relevance score measures the level of similarity of all generated biclusters compared to implanted biclusters. A correlation threshold is required to run BICLIC, BCCA and CPB. Since all biclusters in the simulated datasets are perfectly correlated, 1 is used as the correlation threshold to run BICLIC, BCCA, and CPB. The minimum numbers of rows and columns in biclusters, additional parameters of BICLIC, are set to five in order to filter out excessively small biclusters. We set *RR* parameters in CPB, parameter used for setting reference rows, as −1 in order not to limit the reference gene to a single gene and to find diverse biclusters related to each individual gene. We varied the *r* parameter in QUBIC, a rank parameter to discretize up- or down-regulated genes, from one to three and selected two for the maximum average match score in this experiment. After biclusters are generated with each algorithm, the average recovery and relevance scores are calculated using the match score in equation 7. Mean values of recovery and relevance scores are calculated from 10 independent simulated datasets for each pattern. The values are reported in Tables 1 and 2. Table 1 shows the average recovery score of each biclustering algorithm in each correlated pattern. BICLIC shows a perfect recovery score in every correlated pattern. In contrast, the performances of BCCA are poor in all correlated patterns, although BCCA is known for its ability to extract biclusters with correlated gene expression patterns. It is thought that BCCA cannot extract relatively small sized correlation-based biclusters in a column dimension that is 10% of the entire column size in a data matrix, because BCCA finds correlation-based biclusters with backward elimination of columns. The average recovery score of CPB was 1, 0.996, and 0.915 in the shifting, scaling, and shifting-scaling pattern, respectively. CPB showed good performance in finding the simulated correlated expression pattern. QUBIC performed poorly in regard to correlated datasets, particularly in scaling patterns for recovering implanted biclusters, because it is difficult to capture correlated patterns with a discretized matrix, which is not an up- or down-regulated pattern. The mean values of average relevance scores of biclustering algorithms for each correlated pattern are shown in Table 2. Average relevance scores of BICLIC are 1 in every correlated pattern. This means that all of the extracted biclusters from BICLIC are perfectly related to true implanted biclusters. In contrast, about 90% of extracted biclusters from BCCA and QUBIC are irrelevant to true biclusters. Although the average relevance score of CPB in each correlated pattern was higher than that of BCCA and QUBIC, more than 30% of extracted biclusters from CPB are irrelevant to true biclusters. To test the ability of unbiased search for various sizes of biclusters with correlated patterns, each bicluster algorithm was applied to each pattern of the simulated dataset with varying column fraction level of bicluster size. The average recovery score of each biclustering algorithm in each correlated pattern is shown in Figure 1. BICIC and CPB showed the perfect average recovery score, 1, for all correlated patterns in every column fraction level. CPB could extract obvious correlated expression patterns regardless of column fraction level. Although BCCA can extract true implanted biclusters perfectly when the column fraction level is equal to or greater than 60%, the performance drops sharply when the column fraction level is 20% or 40%. This indicates that BCCA cannot find biclusters with a small columns sizes, because BCCA finds biclusters by backward elimination of columns from all columns in a dataset. In other words, BCCA cannot find diverse sizes of correlation-based biclusters, compared to BICLIC. The average recovery score of QUBIC is less than 1 when the column fraction level is not 100%, for all correlated patterns. Most average recovery scores of QUBIC increase with increasing column fraction level. Because QUBIC drops genes that are not significantly up- or down-regulated during the discretization process, subtly changing correlated patterns of genes in small sized columns cannot be found.

**Table 1:** Comparison of average recovery scores for simulated datasets with various correlated patterns

**Algorithm**	**Shifting**	**Scaling**	**Shifting-Scaling**
BICLIC	1	1	1
BCCA	0.141	0.181	0.168
CPB	1	0.996	0.915
QUBIC	0.431	0.169	0.466

The maximum and minimum numbers of the average recovery score are 1 and 0, respectively. Each average recovery score in Table 1 is the mean value of the average recovery scores from 10 independent datasets.

**Table 2:** Comparison of average relevance scores for simulated datasets with various correlated patterns

**Algorithm**	**Shifting**	**Scaling**	**Shifting-Scaling**
BICLIC	1	1	1
BCCA	0.060	0.109	0.094
CPB	0.143	0.297	0.258
QUBIC	0.038	0.043	0.107

The maximum and minimum numbers of the average recovery scores are 1 and 0, respectively. Each average relevance score in Table 2 is the mean value of average relevance scores from 10 independent datasets.

### Experimental dataset

To investigate the usefulness of BICLIC in searching comprehensive sets of correlation-based biclusters, a yeast Saccharomyces cerevisiae dataset [25] and lung cancer dataset [26] were analyzed. The yeast Saccharomyces cerevisiae dataset shows yeast gene expression under different stress conditions. It consists of 2993 genes and 173 conditions. The lung cancer dataset contains 12,625 genes and 56 samples. 56 samples consists of 20 pulmonary carcinoid samples, 13 colon cancer metastasis samples, 17 normal lung samples, and 6 small cell lung carcinoma samples. Since BICLIC is able to extract biclusters from numeric values of a microarray data matrix, no pre-processing step such as discretization or taking logarithms is necessary for BICLIC analysis. BCCA and CPB also do not require a pre-processing step, but the QUBIC algorithm includes a discretization step. Correlation thresholds for BICLIC, BCCA, and CPB were set to 0.9 to search biclusters with highly correlated expression. The minimum number of rows, *mnr,* and minimum number of columns, *mnc,* parameters of BICLIC were both set to five in order to filter out particularly small biclusters. For CPB, we set the maximum overlap level, MO, to 1 and the maximum number of biclusters, *NB,* to 10,000 to extract comprehensive sets of biclusters. In addition, we did not determine the reference rows of CPB to extract biclusters related with diverse sets of individual genes. We varied the parameters of QUBIC to report more comprehensive sets of biclusters. Although the default value of parameter *o* in QUBIC is 100, restricting the number of biclusters, it was set to 10,000 to report the maximum number of biclusters. Duplicated biclusters are removed in the results of each biclustering algorithm.

Table 3 summarizes the performance of the tested methods for yeast stress datasets. The number of found biclusters after each biclustering method was applied to the yeast stress dataset is reported. Values in parenthesis represent values of seed biclusters using BICLIC. BICLIC found 11,172 seed biclusters, which is greater than the number of biclusters found by BCCA, CPB, and QUBIC. These seed biclusters were expanded to larger correlation-based biclusters, and BICLIC found 14,791 non-duplicated correlation-based biclusters. BCCA, CPB, and QUBIC found 8,163, 3,634, and 2,146 biclusters, respectively, but these numbers are considerably less than that of BICLIC. Therefore, BICLIC searched correlation-based biclusters much more comprehensively. Afterwards, the average sizes of extracted biclusters were computed. After the expanding and filtering steps, an average size of seed biclusters of BICLIC of 7.2, dramatically increased to 2249.3. The biclusters extracted by BCCA and CPB have a larger average size than those extracted by BICLIC and QUBIC. However, most of the extracted biclusters from BCCA and CPB tend to be highly overlapped. QUBIC has the smallest average size of biclusters among the compared methods. To investigate the comprehensiveness of extracted biclusters, coverage was calculated in the gene dimension and the condition dimension for all cells in the dataset. The area covered by extracted biclusters was investigated for a 2993 X 173 data matrix. If at least one bicluster contains a particular gene or condition, that gene or condition is covered with searched biclusters. Cell coverage is calculated in the same way of gene coverage or condition coverage. If a certain cell is included in at least one bicluster, that cell is covered with searched biclusters. The coverage of each algorithm is listed in Table 3. Seed biclusters of BICLIC covered 90.5% of genes, 100% of conditions, and 10.9% of cells in the yeast stress dataset. Moreover, expanded biclusters of BICLIC covered 100% of genes, 100% of conditions, and 99.9% of cells. In other words, BICLIC found a comprehensive set of correlation-based biclusters and most genes and conditions in datasets were included in at least one bicluster. Compared to that, searched biclusters from BCCA only covered 77.6% of genes and 31.7% of cells in the data matrix although 100% of conditions were covered by biclusters. Even though BCCA extracted 8,163 biclusters with the greatest average size, those biclusters covered only a small fraction of cells in the yeast stress data matrix. In other words, BCCA cannot search diverse sets of biclusters, and most searched biclusters in BCCA are highly overlapped. CPB also searched highly overlapped biclusters. Although average size of biclusters in CPB was 8413.6, those biclusters only cover 51.2% of genes and 18.5% of cells in the data matrix. The searched biclusters from QUBIC covered the smallest fraction of cells among the compared methods. This means that the discretization step hinders the search for a diverse set of biclusters. Only highly up-regulated or down-regulated genes in limited conditions, which are about 11.2% of cells in a data matrix, are searched in QUBIC.

**Table 3:** Summary statistics of biclustering algorithms for the yeast stress dataset

**Method**	**Count**	**Average |I x J|**	**Gene cov.**	**Condition cov.**	**Cell cov.**
BICLIC	14791	2249.3	1	1	0.999
	(11172)	(7.2)	(0.905)	(1)	(0.109)
BCCA	8163	2936.8	0.776	1	0.317
CPB	3634	8413.6	0.512	1	0.185
QUBIC	2146	847.4	0.884	0.746	0.112

Values in parentheses denote the values of seed biclusters of BICLIC. The columns “Count”, “Average |I x J|”, “Gene cov.”, “Condition cov.”, and “Cell cov.” show the numbers of biclusters, average sizes of biclusters, coverage of biclusters in the gene dimension, coverage of biclusters in the condition dimension, and coverage of biclusters for all cells in the matrix.

Table 4 summarizes the performance of the tested methods for lung cancer datasets. BCCA was eliminated for this test, because conducting BCCA could not be completed with this dataset in reasonable time. The number of bicluster found by the three biclustering algorithms, BICLIC, CPB, and QUBIC, was reported. BICLIC found 6,019 non-duplicated correlation-based biclusters. CPB and QUBIC found 386 and 1,355 biclusters, respectively. Particularly, CPB found considerably small number of biclusters. It means that it is inadequate to use CPB in finding diverse sets of biclusters despite the fact that CPB may perform well in finding correlated patterns of biclusters that are related with reference genes. The average size of biclusters found by CPB was 4,594.8, but the cell coverage was only 0.344. It indicates that a number of genes and conditions of biclusters found by CPB may be highly overlapped among themselves. BICLIC covered 100% of genes, 100% of conditions, and 99.9% of cells. In other words, BICLIC found diverse sets of biclusters with correlated expression patterns for lung cancer dataset.

**Table 4:** Summary statistics of biclustering algorithms for the lung cancer dataset

**Method**	**Count**	**Average |I x J|**	**Gene cov.**	**Condition cov.**	**Cell cov.**
BICLIC	6019	2302.8	1	1	0.999
	(3734)	(4.2)	(0.389)	(1)	(0.021)
CPB	386	4594.8	0.672	1	0.344
QUBIC	1355	68.2	0.543	1	0.048

Values in parentheses denote the values of seed biclusters of BICLIC. The columns “Count”, “Average |I x J|”, “Gene cov.”, “Condition cov.”, and “Cell cov.” show the numbers of biclusters, average sizes of biclusters, coverage of biclusters in the gene dimension, coverage of biclusters in the condition dimension, and coverage of biclusters for all cells in the matrix.

**Figure 2:**
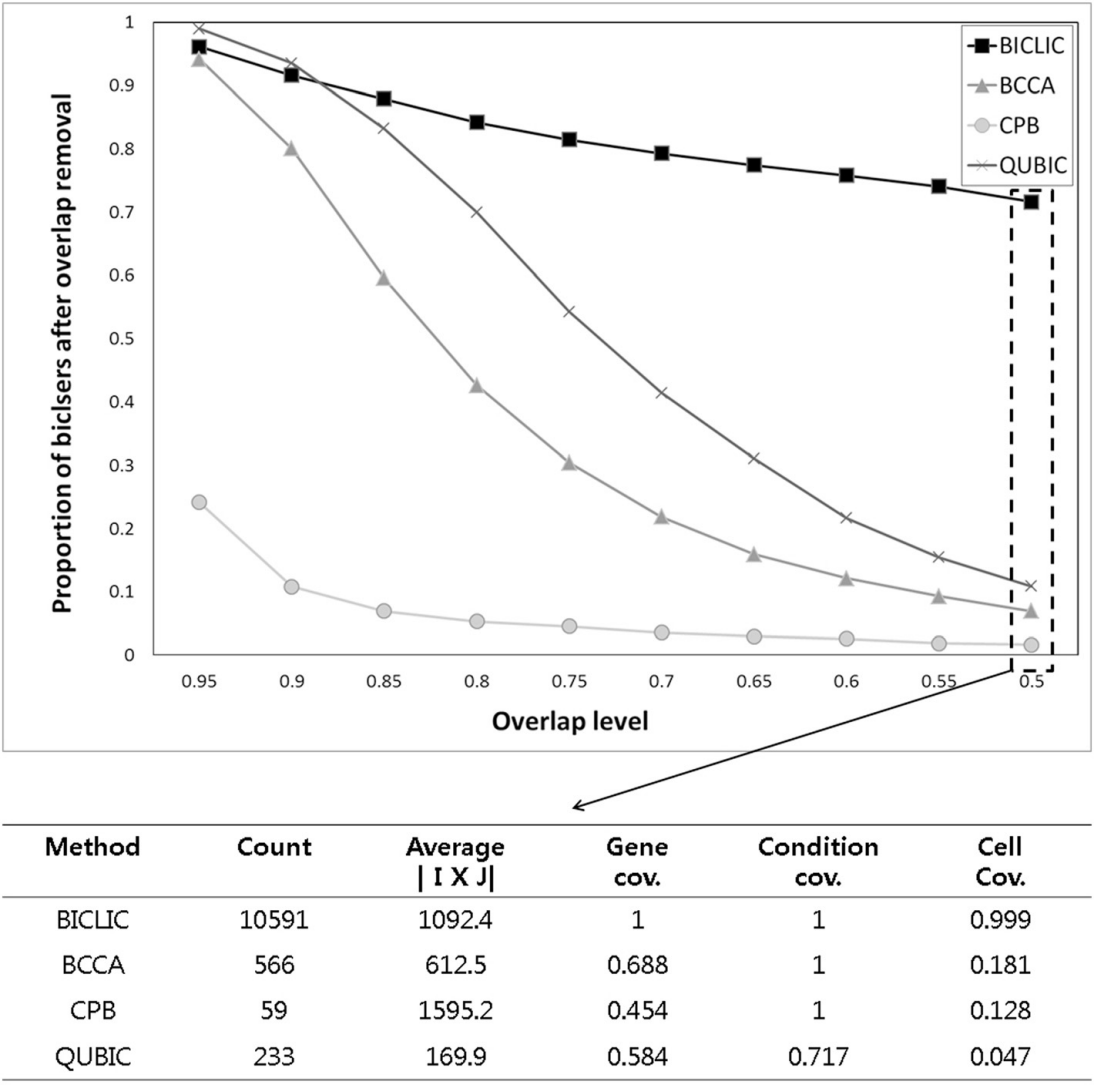
Proportion of the remaining biclusters after removing overlapping biclusters in each biclustering algorithm for yeast stress dataset.

In an additional experiment, the overlap level of extracted biclusters in yeast stress dataset was evaluated for BICLIC, BCCA, CPB, and QUBIC. All searched biclusters for each biclustering algorithm were arranged in decreasing order of bicluster size. When a bicluster had *o*% of its cells in common with any larger size biclusters, that bicluster was filtered. The remaining number of biclusters was computed after filtering overlapped biclusters with *o*% of overlap level. The proportions of biclusters remaining after removing overlapped biclusters by varying the overlap level for each biclustering algorithm are shown in Figure 2. In addition, summary statistics of the biclustering algorithm after removing overlapped biclusters at the 50% overlap level are presented in Figure 2. Details about summary statistics at other overlap levels are provided in Additional file 1: Table S1. The proportion of biclusters remaining decreased as the overlap level, the threshold at which to filter overlapped biclusters, decreased. However, the slope of decreasing proportions varies in each biclustering algorithm. While the proportion of biclusters remaining in BICLIC decreased slowly, that in BCCA, CPB, and QUBIC decreased rapidly. If biclusters that have an 80% overlap level with larger biclusters are filtered, only 5.3% of biclusters in CPB remain. 84% of biclusters in BICLIC remain on the same overlap level. Moreover, 71% of biclusters in BICLIC remain, but only 6.9% and 1.6% of biclusters were remained in BCCA and CPB, respectively when overlapped biclusters with 50% overlap level were filtered. At this overlap level, the average size of searched biclusters in BCCA is 612.5, which is much smaller than the average size of 1092.4 in BICLIC. In addition, only 233 biclusters are left in QUBIC after removing smaller sized biclusters that have more than 50% cells in common with any other larger biclusters. These remaining biclusters cover only 4.7% of cells in the data matrix. This means that BICLIC extracted more comprehensive and not highly overlapped sets of bicluster than BCCA, CPB, and QUBIC.

### Function enrichment evaluation

**Figure 3:**
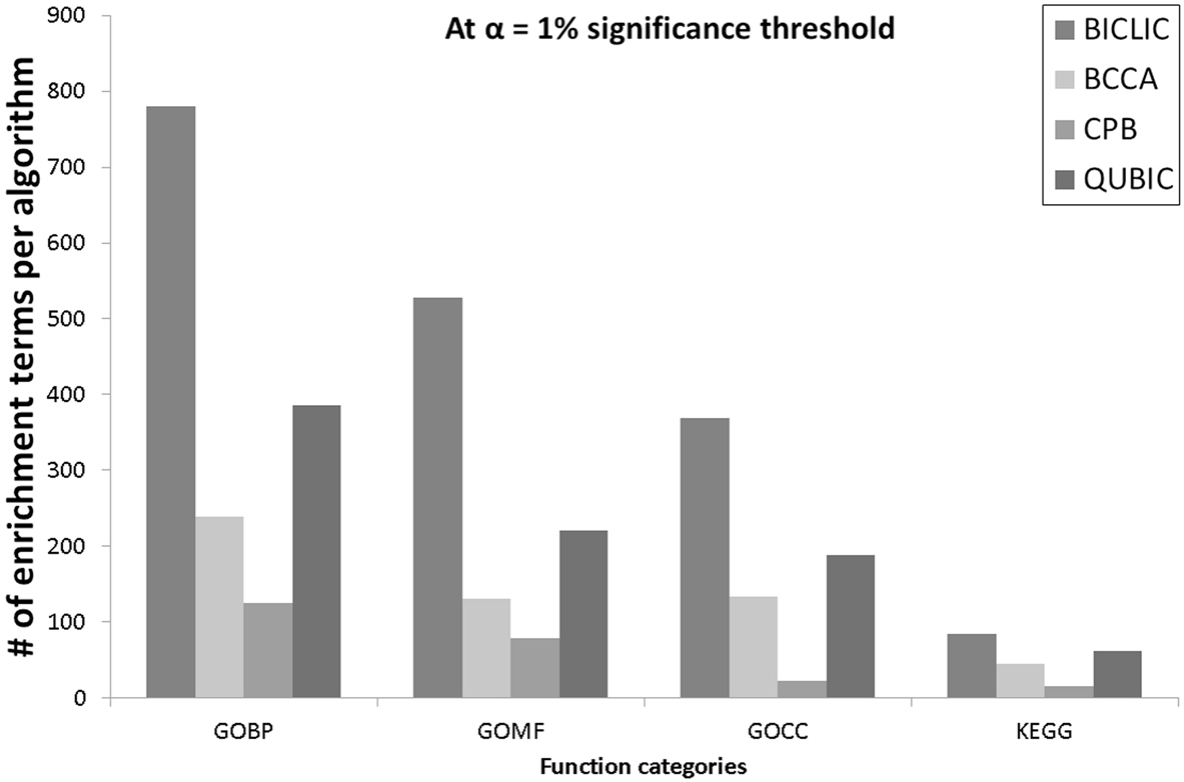
The number of significantly enriched biological terms for four bi-clustering algorithms in four functional categories at 1% significance threshold for yeast stress data set.

To investigate the biological relevance of extracted biclusters, functional enrichment of extracted biclusters was conducted with the GO functional category and the KEGG biological pathway for each biclustering algorithm. A modified version of COFECO (composite function annotation enriched by protein complex data) was used for functional enrichment analysis [27]. All searched biclusters from each biclustering algorithm were enriched to four functional categories: GO biological process (GO BP), GO molecular function (GO MF), GO cellular component (GO CC), and KEGG pathway (KEGG). The significance of association between a set of genes in a bicluster and a functional term was estimated by a hypergeometric test. The false discovery rate (FDR) multiple-testing correction [28] technique was applied to the estimated p-values in order to avoid the situation whereby the higher the number of genes included in a bicluster, the more significant will be the p-value of the function enrichment. To test the ability to extract a comprehensive set of functional terms, the number of enrichment terms was calculated under the given significance threshold. Among identical functional enrichment terms, the term with the highest significance p-value level was regarded as the unique one. In order to select it, the terms that had a larger significance p-value level were removed, so that the most significant term remained. Figure 3 shows the number of enriched functional terms for searched biclusters in each functional category on the 1% significance level for yeast stress dataset. Details of the number of enriched functional terms with a variety of significance levels are provided in Additional file 1: Figure S1. Function enrichment results appear to be similar in all functional categories.

BICLIC found the largest number of significantly enriched functional terms compared to BCCA, CPB, and QUBIC in GO BP, GO CC, GO MF, and KEGG. Compared to QUBIC, BCCA and CPB found fewer unique functional terms, despite the fact that BCCA and CPB found more and larger biclusters. This means that there are a number of highly overlapped genes and conditions in the biclusters found by BCCA and CPB. Furthermore, the functional enriched terms are also highly redundant in BCCA and CPB. In contrast, BICLIC found comprehensive sets of biclusters. Moreover, it could obtain a number of significant results from the functional enrichment process with GO BP, GO CC, GO MF, and KEGG.

**Figure 4:**
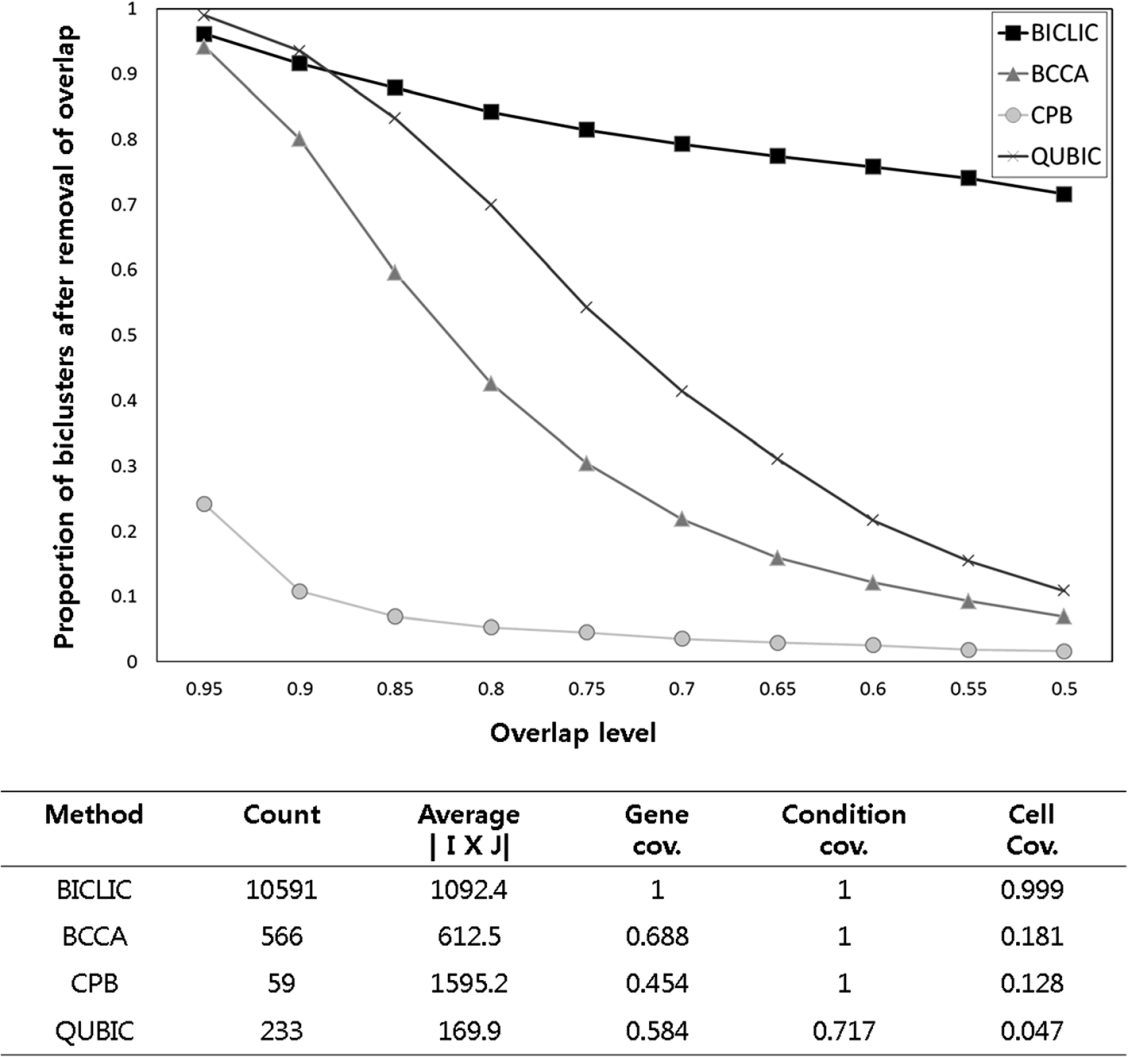
The number of significantly enriched biological terms for three bi-clustering algorithms in four functional categories at 1% significance threshold for lung cancer data set.

We also conducted functional enrichment of extracted biclusters in the lung cancer dataset with the same way of analysing the yeast stress dataset mentioned above. Figure 4 shows the number of enriched functional terms for extracted biclusters of BICLIC, CPB, and QUBIC in four functional categories on the 1% significance level. The tendency shown in the lung cancer dataset is similar to that shown in the yeast stress dataset. BICLIC found the largest number of significantly enriched functional terms compared to CPB and QUBIC. The small number of uniquely enriched terms in CPB algorithm results in finding only small number of biclusters.

## Conclusions

In this paper, we proposed a novel biclustering method, BICLIC, to search for comprehensive sets of correlation-based biclusters. Our algorithm conducts individual dimension-based clustering for efficient determination of comprehensive sets of correlated seed biclusters, which are further expanded to larger correlation-based biclusters. Simulated and real microarray datasets were used to perform several experiments, and the results were compared to those obtained using BCCA, CPB, and QUBIC. The experiments showed that BICLIC could find implanted correlated biclusters accurately while other competing methods such as BCCA and QUBIC performed poorly. In addition, BICLIC was able to extract more comprehensive sets of biclusters than other biclustering algorithms. Although CPB performed well in the simulated dataset, it performed poorly in the real microarray datasets. Finally, the biclusters searched by BICLIC could be enriched to more diverse biological terms in GO and KEGG.

## Methods

**Figure 5:**
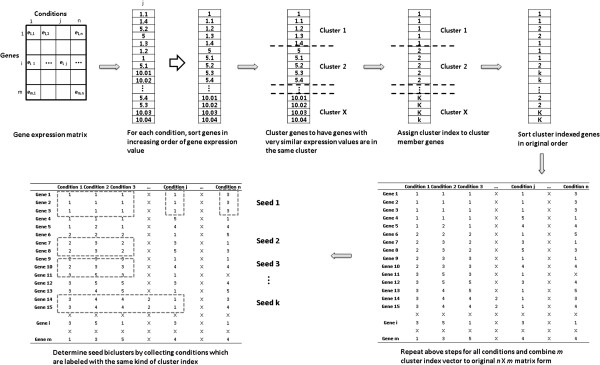
Schematic diagram of determining seed biclusters.

**Figure 6:**
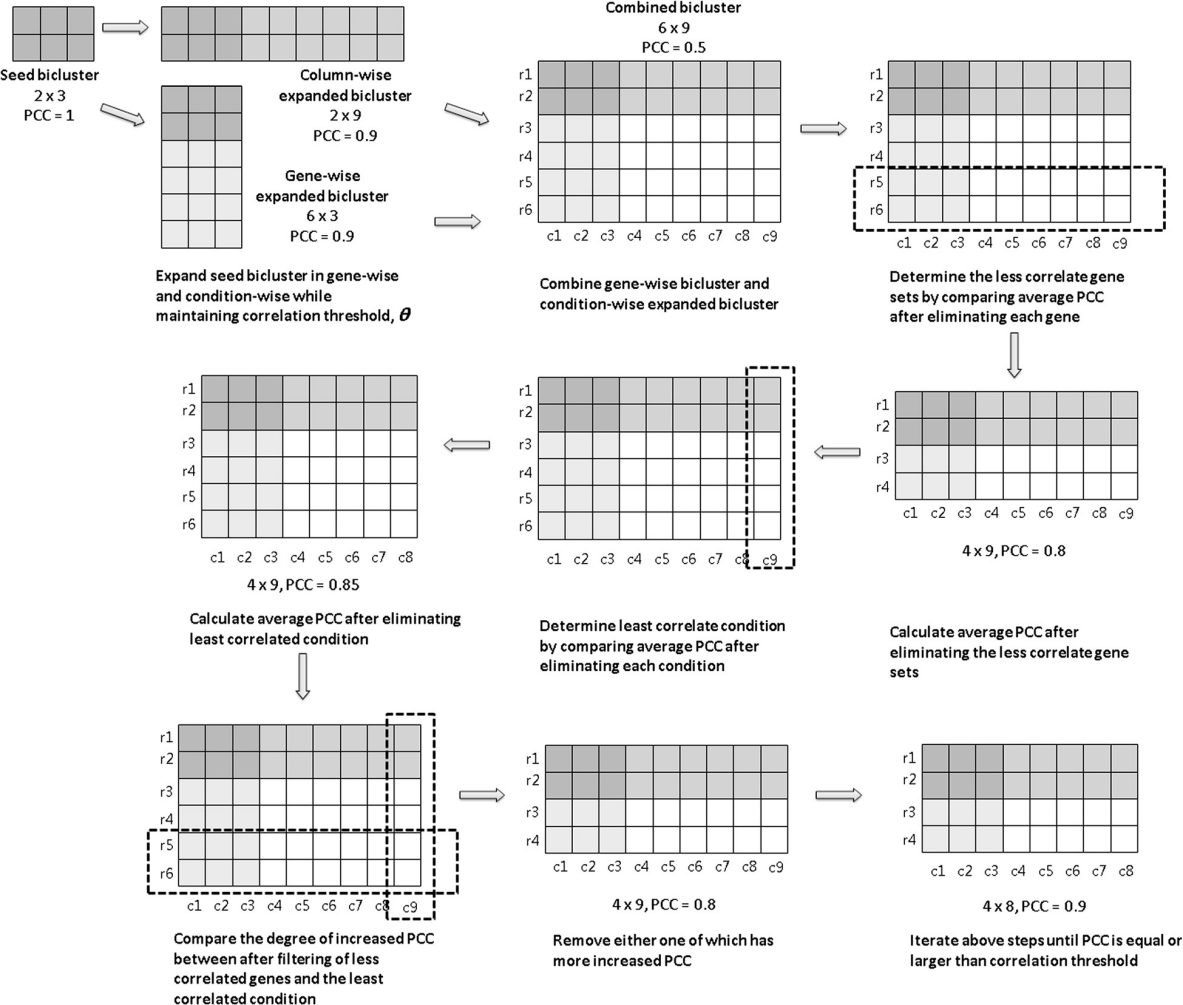
Schematic diagram of expanding seed biclusters.

BICLIC biclustering method consists of four phases: finding comprehensive seed biclusters, expanding seed biclusters, filtering less correlated genes and conditions, and checking and removing duplicated biclusters. The process of finding comprehensive seed biclusters is summarized at Figure 5. The process of expanding seed biclusters and filtering less correlated genes and conditions is summarized at Figure 6. The input parameters are a gene expression matrix, *E*, the correlation threshold value, *θ*, the minimum number of rows, *mnr*, and the minimum number of columns, *mnc*.

### Definitions

#### Definition 1

An input microarray matrix, *E(G,C)*, is *defined* as an *n* X *m* matrix of real numbers, where *G* = {*g*_*1*_*, g*_*2*_*, …g*_*i*_*, …, g*_*n-1*_*, g*_*n*_} is a set of genes and C = {*c*_*1*_, *c*_*2*_, …, *c*_*j*_, … *c*_*m-1*_, *c*_*m*_} is a set of conditions.

#### Definition 2

A seed bicluster, *SB*(*G’*, *C’*), is a small bicluster that is a candidate for being expanded to a larger bicluster, with *G’* ⊆ *G* and *C’* ⊆ *C*. Sets of genes in each condition have the same cluster index, which is generated from individual dimension-based clustering for each condition. In other words, the gene expression values of genes in the same condition in a seed bicluster are very close to each other. Genes across a set of conditions in a seed bicluster show a correlated expression pattern. Therefore, each seed bicluster has two characteristics: an identical or very similar gene expression value in each condition, and a highly correlated gene expression pattern across conditions.

#### Definition 3

An expanded bicluster, *BC*, means that it is expanded from a seed bicluster to have larger elements of genes and conditions while maintaining an average Pearson correlation coefficient above a correlation threshold *θ*. Seed biclusters can be expanded in two directions: gene-wise and condition-wise.

### Finding comprehensive seed biclusters

The generation of seed biclusters is illustrated in Algorithm 1. In this phase, comprehensive sets of initial biclusters are to be found and they will be expanded in a later phase. This phase consists of two steps: individual dimension-based clustering and seed bicluster determination. An *n* X *m* microarray matrix can be decomposed into *m* separate n X 1 vectors. Individual dimension-based clustering is employed to collect genes with similar expression levels in each decomposed vectors. It is an approach that is similar to that used in the Clustering analysis of Large microarray datasets with Individual dimension-based Clustering (CLIC) algorithm [29]. CLIC uses individual dimension-based clustering method to cluster larger microarray datasets efficiently. In this paper, individual dimension-based clustering is conducted for *n* genes in each array of a dimension to divide very similarly expressed genes that are in the same cluster in one dimension. That is, thousands of genes in each condition are clustered into a large number of small sized clusters that contain highly similarly expressed genes.

An individual dimension-based clustering method is more efficient than those conventional approaches although conventional clustering algorithms such as *k*-means and hierarchical clustering can be used. *K*-means clustering requires additional steps to determine the appropriate number of clusters in each dimension, and hierarchical clustering needs to calculate the distances between all pairs of genes. Therefore, we used the following individual dimension-based clustering approach to clusters efficiently genes with very similar expression in each dimension. Threshold values to determine whether the genes should be selected in each cluster in each condition are standard deviations of whole gene expression values in each condition and a cumulative standard deviation of gene expression values (in Step 1C and 1Ed).

After individual dimension-based clustering, the genes that have similar expression values in each individual condition are labeled with the same cluster index. The *m* cluster index vectors are recombined into the original *n* X *m* matrix form. Comprehensive seed biclusters are determined from this cluster index matrix. The sum of the numbers of clusters that are determined in individual dimension-based clustering over all conditions indicates the number of candidate seed biclusters. The number of discovered seed biclusters is sufficient because genes with similar expression in each condition are very finely divided to have a large number of biclusters. Genes in candidate seed biclusters in each condition are labeled with the same cluster index. These genes in another condition can be labeled with either different or the same kinds of cluster indexes. If genes in another condition are labeled with the same kind of cluster index, it means that the gene expression levels are similar not only in the original condition but also in the other conditions. In other words, genes show correlated expression patterns over these conditions. Non-duplicated sets of diverse seed biclusters are determined in this phase. These seed biclusters are more correlated than randomly extracted seed biclusters. Moreover, the same seed biclusters can be determined even in multiple executions of the algorithm.

### Algorithm 1 Seed Bicluster Extraction Algorithm

**Input:***E*: *n* X *m* gene expression matrix

**Output:***SB*: List of seed biclusters

Steps:

1. Individual dimension-based clustering

For each m individual condition, do:

A. Align gene set *G*={*g*_*1*_,*g*_*2*_,…*g*_*n*_} to *G`*={*g*_*1*_‘,*g*_*2*_‘,..,*g*_*n*_‘ } in increasing order of gene expression value, where *g*_*1*_‘ ≤ *g*_*2*_*‘* ≤, … , ≤ *g*_*n-1*_‘ ≤ *g*_*n*_‘.

B. Initially, set gene index *i* = 1 and set cluster index *KI* to 1.

C. Measure standard deviation of all genes in this condition and set it as *sd_all*.

D. Let *K*_*KI*_ for set of cluster member genes when cluster index is *KI* and set *K*_*KI*_ ==NULL

a. Set cumulative number of genes in cluster set, *cum* = 0

b. *K*_*KI*_ = *K*_*KI*_ ∪ {*g*_*i*_‘ }.

c. Assign cluster index *KI* to cluster member gene.

E. If cluster *K*_*KI*_ !=NULL, then

a. Set *cum* = *cum* + 1

b. Set *i* = *i* + 1.

c. Set *K*_*KI*_ = *K*_*KI*_ ∪ {*g*_*i*_‘ }.

d. Measure standard deviation of *K*_*KI*_ when number of member gene in cluster set is *cum*, sd(*K*_*KI, cum*_).

e. While sd(*K*_*KI, cum*_) ≤ sd(*K*_*KI, cum-1*_) and sd(*K*_*KI, cum*_) ≤ *sd*_*all*, do:

i. Set *i* = *i* + 1.

ii. Set *K*_*KI*_ = *K*_*KI*_ ∪ {*g*_*i*_’ }.

iii. Assign cluster index *KI* to cluster member genes.

f. If sd(*K*_*KI, cum*_) > sd(*K*_*KI, cum-1*_) or sd(*K*_*KI, cum*_) >*sd_all*.

i. Set *KI* = *KI* + 1.

ii. Set *K*_*KI*_ = *K*_*KI*_ ∪ { *g*_*i*_’ }.

iii. Assign cluster index *KI* to cluster member genes.

iv. Set *cum* = 0.

F. Repeat Step 1D to 1E until *i* == *n*.

G. Align cluster indexed genes i.e. {1, 1, 2, 2, …, *KI* – 2, *KI* - 1, *KI*} to original order as in *G*={*g*_*1*_,*g*_*2*_,…*g*_*n*_}.

H. Combine m cluster index vector to original *n* X *m* matrix form.

2. Seed bicluster determination

For each m individual condition, do:

A. Initially, Set seed bicluster set *S* = NULL

B. For *s* = 1 to *KI* in each condition, do:

a. Let *g*(*K*_*s*_) for rows of genes when cluster index *KI* is s in each condition.

b. Set seed cluster condition set, *CS* = NULL.

c. For *j* = 1 to *m* condition, do:

i. Let *g*(*K*_*s, j*_) for the collection of genes when genes are in *g*(*K*_*s*_) rows and condition is in *j*th column.

ii. If genes in *g*(*K*_*s, j*_) have same kinds of cluster index, then set *CS* = *CS* ∪ {*c*_*j*_}

iii. If the number of elements in *CS* ≥ 2

-Set seed bicluster, *sb*, consist of *g*(*K*_*s*_) and *CS*

-Add each seed bicluster, *sb* to seed bicluster list, *SB*

Expanding seed biclusters

In this phase, previously determined comprehensive sets of seed biclusters are expanded to larger biclusters with correlated patterns. The Pearson correlation coefficient is used as scoring function to measure correlation between pairs of genes over subsets of conditions when seed biclusters are expanded, while maintaining similarity over a correlation threshold. BICLIC uses a heuristic approach to expand seed biclusters efficiently by merging each gene or each condition from the most similar one to the least similar one with a seed bicluster. Each seed bicluster is expanded in two ways, gene-wise and condition-wise, while maintaining the average Pearson correlation coefficient of pairs of genes over conditions in each expanded bicluster above the correlation threshold. The computation required in this heuristic approach is considerably less than that in the approach of exhaustive search of all possible combinations of genes and conditions. Although less comprehensiveness in the expanded biclusters may appear in the proposed heuristic approach than in an iterative approach, this disadvantage can be alleviated by the existence of comprehensive sets of correlated seed biclusters.

In gene-wise expansion, the minimum number of conditions in seed biclusters must be equal to or greater than 3. Otherwise, the average Pearson correlation coefficient of gene-wise expanded biclusters will be +1, -1, or non-computable. For each seed bicluster, the Pearson correlation coefficient value between a seed bicluster and each gene vector is calculated to find candidate sets of correlated genes to expand. Then, each gene is merged to a seed bicluster in decreasing order of correlation coefficients between gene vectors and the seed bicluster to add similar genes to the seed bicluster efficiently, until the average Pearson correlation coefficient of the gene-wise expanded biclusters is no longer smaller than the correlation threshold value, θ. Such an efficient gene expansion approach also leads to stable expansion results because the order of genes to expand is determined when calculating the Pearson correlation coefficient value between a seed bicluster and each gene vector. The Pearson correlation coefficient between a seed bicluster and a gene vector is calculated using equation 1.

*SB*_*mean*_ is the mean expression vector of a seed bicluster and *gv*_*i*_ is the *i*th gene expression vector that has the same column dimension as *SB*.

(1) $$\mathrm{Corr}\left(SB_{\mathrm{mean}},gv_{i}\right)=\frac{\sum_{l=1}^{m'}\left(SB_{\mathrm{mean},l}-{SB}_{\mathrm{mean}}^{¯}\right)\left(gv_{i,l}-{g\bar{v}}_{i}\right)}{\sqrt{\sum_{l=1}^{m'}{\left(SB_{\mathrm{mean},l}-{SB}_{\mathrm{mean}}^{¯}\right)}^{2}\sqrt{{\sum_{l=1}^{m'}\left(gv_{i,l}-{g\bar{v}}_{i}\right)}^{2}}}}$$

In condition-wise expansion, the correlation coefficient of an expanded seed bicluster is computed when each candidate condition is merged to a seed bicluster. Condition-wise expansion checks whether genes in a seed bicluster have additional correlated expression patterns in the remaining conditions. If the average correlation coefficient of a condition-wise expanded bicluster is greater than the correlation threshold, genes in such biclusters show a correlated expression pattern over both conditions in the seed bicluster and expanded conditions. The average Pearson correlation coefficient of biclusters after expanding condition *j* is defined in equation 2.

(2) $$\begin{array}{l} \begin{array}{l} cor_{j}=\frac{1}{{}_{n}C_{2}}\sum_{p=1}^{n-1}\sum_{q=p+1}^{n}\mathrm{Corr}\left(\mathrm{tmpC}E_{g_{p}},\mathrm{tmpC}E_{g_{q}}\right)\\ where \end{array}\\ \mathrm{Corr}\left(\mathrm{tmpC}E_{g_{p}},\mathrm{tmpC}E_{g_{q}}\right)\\ =\frac{\sum_{l=1}^{m'+1}\left(\mathrm{tmpC}E_{g_{p,l}}-\mathrm{tm}\overset{-}{\mathrm{pC}}E_{g_{p}}\right)\left(\mathrm{tmpC}E_{g_{q},l}-\mathrm{tm}\overset{-}{\mathrm{pC}}E_{g_{p}}\right)}{\sqrt{{\sum_{l=1}^{m'+1}\left(\mathrm{tmpC}E_{g_{p,l}}-\mathrm{tm}\overset{-}{\mathrm{pC}}E_{g_{p}}\right)}^{2}\sqrt{\sum_{l=1}^{m'+1}{\left(\mathrm{tmpC}E_{g_{q},l}-\mathrm{tmpC}E_{g_{q}}\right)}^{2}}}} \end{array}$$

If *cor*_*j*_ is greater than the overall correlation threshold *θ*, then all of the genes in the bicluster are still highly correlated, after condition *j* is added. Conditions are merged to a seed bicluster in decreasing order of Pearson correlation coefficients of expanded biclusters to add similar conditions to a seed bicluster efficiently until the correlation coefficient of a condition-wise expanded bicluster is not less than the correlation threshold, *θ.* This condition-wise expansion approach also leads to stable expansion results, because the order of conditions to expand is determined by the value of the Pearson correlation coefficient.

After expanding a seed bicluster in gene-wise and condition-wise directions, a vertically and horizontally long matrix can be acquired, respectively. These two matrices can be combined to form a larger matrix that has rows in the gene-wise expanded bicluster and columns in the condition-wise expanded bicluster. This combined matrix is theoretically the largest size of matrix to which a seed bicluster can be expanded. The correlation coefficient of this matrix is less than the correlation threshold *θ* because not all genes are correlated under a set of conditions in the combined matrix. By filtering uncorrelated genes and conditions in this combined matrix, a large bicluster with correlated pattern can be acquired. Gene-wise and condition-wise expanded biclusters are also candidate correlation-based biclusters that BICLIC algorithm has found.

### Filtering less correlated genes and conditions

Each correlated seed bicluster is enlarged to a larger candidate bicluster by combining gene-wise expanded biclusters and condition-wise expanded biclusters. Although not all genes may show correlated patterns over all conditions in a candidate bicluster matrix, at least all genes and conditions in this candidate bicluster are correlated with the seed bicluster. Correlation-based biclusters can be acquired by backwardly eliminating less correlated sets of genes and conditions. Algorithm 2 illustrates the steps of filtering less correlated genes and conditions. The average Pearson correlation coefficient, *θ*_*CB*_*,* is the average value of the Pearson correlation coefficient for all pairs of genes over conditions in candidate biclusters. *θ*_*CB*_ is defined in equation 3. The vectors g_p_ and g_q_ are the *p*th and *q*th gene expression vector in candidate bicluster *CB*, respectively.

(3) $$\begin{array}{l} \begin{array}{l} \theta_{\mathrm{CB}}=\frac{1}{{}_{n'}C_{2}}\sum_{p=1}^{n'-1}\sum_{q=p+1}^{n'}\mathrm{Corr}\left(g_{p},g_{q}\right)\\ where \end{array}\\ \mathrm{Corr}\left(g_{p},g_{q}\right)=\frac{\sum_{l=1}^{m'}(g_{p,l}-{\bar{g}}_{q})\left(g_{q,l}-{\bar{g}}_{q}\right)}{\sqrt{\sum_{l=1}^{m'}{\left(g_{q,l}-{\bar{g}}_{q}\right)}^{2}}\sqrt{\sum_{l=1}^{m'}{\left(g_{q,l}-{\bar{g}}_{q}\right)}^{2}}} \end{array}$$

In each iteration, the less correlated set of genes and the least correlated condition are calculated from a candidate bicluster matrix. The least correlation condition is eliminated, and then, the degree of increase in the average Pearson correlation coefficient (APCC) of the remaining matrix is measured. While the former result is set aside, in turn, less correlated set of genes of the original matrix are to be eliminated. The degree of increase in the APCC is measured. Then, the two degrees of increased APCC from the previous steps are compared to eliminate the one that has higher degree. For instance, when the degree of increase in the APCC of the least correlation condition is higher than that of less correlated set of genes, the former is eliminated and the latter is remained and vice versa. The number of conditions represents the length of a correlated expression pattern. Therefore, the least correlated condition is compared to a set of less correlated genes to extract a large correlated expression pattern. After removing less correlated sets of genes or the least correlated condition in a repeated way until the average correlation coefficient of the matrix is equal to or greater than the correlation threshold, a correlation-based bicluster matrix is acquired.

## Competing interests

The authors declare that they have no competing interests.

## Authors’ contributions

TY wrote the R-based algorithm, performed experiments, and wrote the manuscript. GSY conceived and supervised this study, and reviewed the manuscript. All authors read and approved the final manuscript.

## Supplementary Material

Additional file 1: Table S1 Summary statistics of remaining biclusters dataset after removing overlapped biclusters by varying the overlap level for the yeast stress in each biclustering algorithm. **Figure S1**. The number of significantly enriched biological terms for thee biclustering algorithms in four functional categories on various significance levels. (a) GO Biological Process, (b) GO Cellular Component, (c) GO Molecular Function, (d) KEGG Pathway. Click here for file
