# Supplementary material for: Biclustering for the comprehensive search of correlated gene expression patterns using clustered seed expansion
Source: BMC Genomics. 2013 Mar 5;14:144. doi: 10.1186/1471-2164-14-144 (PMC3618306; doi:10.1186/1471-2164-14-144)
Supplement: Additional file 1: Table S1 — Summary statistics of remaining biclusters dataset after removing overlapped biclusters by varying the overlap level for the yeast stress in each biclustering algorithm. Figure S1. The number of significantly enriched biological terms for thee biclustering algorithms in four functional categories on various significance levels. (a) GO Biological Process, (b) GO Cellular Component, (c) GO Molecular Function, (d) KEGG Pathway. [file 1471-2164-14-144-S1.docx]

**Biclustering for the comprehensive search of correlated gene expression patterns using clustered seed expansion**

**Supplementary Material**

Table S1. Summary statistics of remaining biclusters dataset after removing overlapped biclusters by varying the overlap level for the yeast stress in each biclustering algorithms

| Algorithm | Overlap level | 0.95. | 0.9 | 0.85 | 0.8 | 0.75 | 0.7 | 0.65 | 0.6 | 0.55 | 0.5 |
| --- | --- | --- | --- | --- | --- | --- | --- | --- | --- | --- | --- |
| BICLIC | Count | 14214 | 13549 | 12997 | 12453 | 12048 | 11728 | 11452 | 11210 | 10948 | 10591 |
|  | Average \|I x J\| | 1950.0 | 1608.3 | 1381.6 | 1249.3 | 1171.1 | 1137.6 | 1124.9 | 1129.6 | 1112.8 | 1092.4 |
|  | Gene  cov. | 1 | 1 | 1 | 1 | 1 | 1 | 1 | 1 | 1 | 1 |
|  | Condition cov. | 1 | 1 | 1 | 1 | 1 | 1 | 1 | 1 | 1 | 1 |
|  | Cell cov. | 0.999 | 0.999 | 0.999 | 0.999 | 0.999 | 0.999 | 0.999 | 0.999 | 0.999 | 0.999 |
| BCCA | Count | 7686 | 6532 | 4863 | 3477 | 2481 | 1782 | 1300 | 988 | 755 | 566 |
|  | Average \|I x J\| | 2734.5 | 2408.6 | 1932.2 | 1623.6 | 1401.2 | 1223.6 | 1028.8 | 881.7 | 749.6 | 612.5 |
|  | Gene  cov. | 0.776 | 0.773 | 0.762 | 0.747 | 0.729 | 0.717 | 0.708 | 0.701 | 0.695 | 0.688 |
|  | Condition cov. | 1 | 1 | 1 | 1 | 1 | 1 | 1 | 1 | 1 | 1 |
|  | Cell cov. | 0.317 | 0.312 | 0.296 | 0.274 | 0.252 | 0.236 | 0.222 | 0.210 | 0.197 | 0.181 |
| CPB | Count | 876 | 392 | 252 | 191 | 164 | 129 | 107 | 92 | 68 | 59 |
|  | Average \|I x J\| | 5649.8 | 3769.7 | 2833.6 | 2305.8 | 2127.2 | 1964.1 | 1891.7 | 1807.3 | 1620.9 | 1595.2 |
|  | Gene  cov. | 0.512 | 0.512 | 0.510 | 0.510 | 0.508 | 0.505 | 0.491 | 0.485 | 0.468 | 0.454 |
|  | Condition cov. | 1 | 1 | 1 | 1 | 1 | 1 | 1 | 1 | 1 | 1 |
|  | Cell cov. | 0.184 | 0.181 | 0.177 | 0.171 | 0.167 | 0.159 | 0.154 | 0.148 | 0.133 | 0.128 |
| QUBIC | Count | 2123 | 2006 | 1785 | 1501 | 1164 | 889 | 666 | 464 | 331 | 233 |
|  | Average \|I x J\| | 839.8 | 800.9 | 714.5 | 617.8 | 510.4 | 420.1 | 347.3 | 268.5 | 200.3 | 169.9 |
|  | Gene  cov. | 0.884 | 0.880 | 0.873 | 0.859 | 0.834 | 0.794 | 0.753 | 0.710 | 0.651 | 0.584 |
|  | Condition cov. | 0.746 | 0.746 | 0.746 | 0.746 | 0.740 | 0.740 | 0.740 | 0.728 | 0.722 | 0.717 |
|  | Cell cov. | 0.112 | 0.111 | 0.109 | 0.106 | 0.010 | 0.092 | 0.084 | 0.073 | 0.059 | 0.047 |

The columns “Count”, “Average |I x J|”, “Gene cov.”, “Condition cov.”, and “Cell cov.” show the numbers of biclusters, average sizes of biclusters, coverage of biclusters in the gene dimension, coverage of biclusters in the condition dimension, and coverage of biclusters for all cells in the matrix.


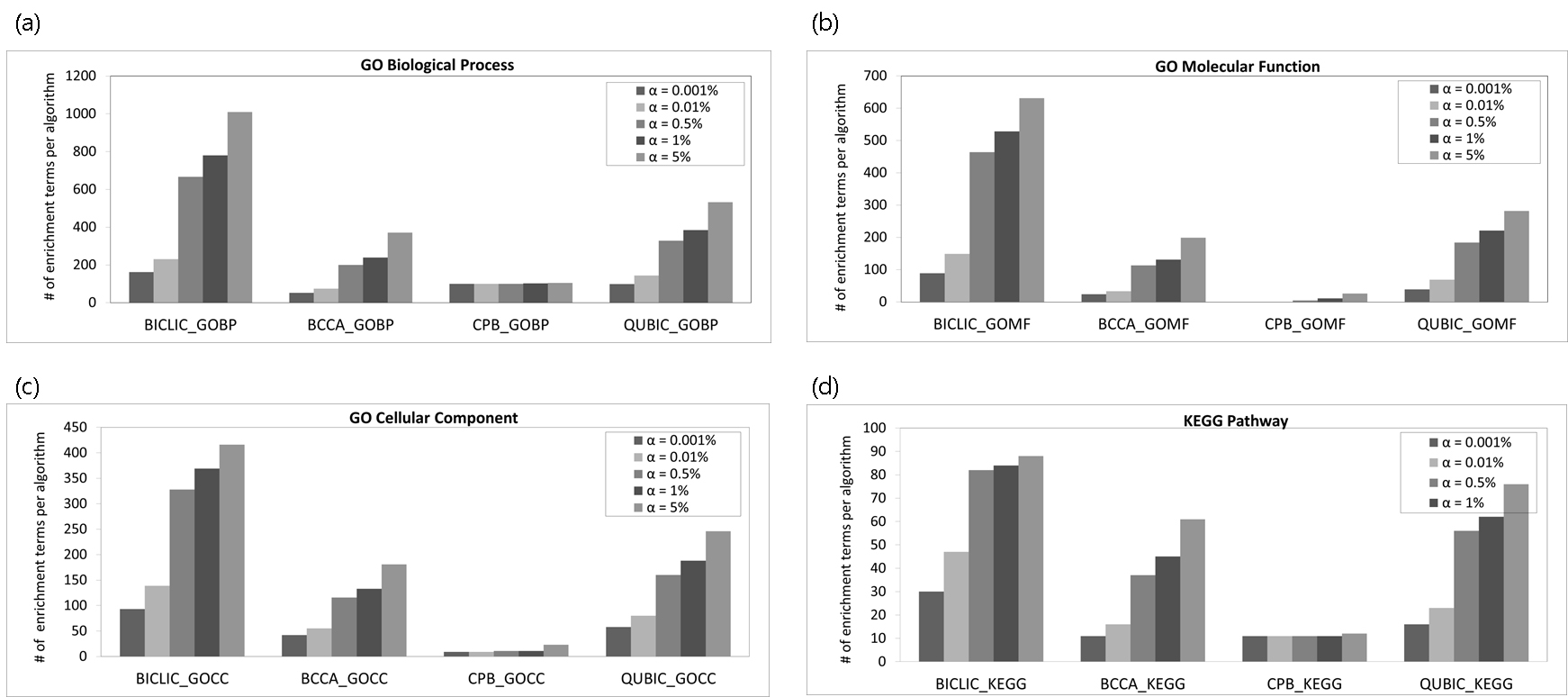


Figure S1. The number of significantly enriched biological terms for four biclustering algorithms in four functional categories on various significance level. (a) GO Biological Process, (b) GO Cellular Component, (c) GO Molecular Function, (d) KEGG Pathway
